# Evaluation of glomerular sirtuin-1 and claudin-1 in the pathophysiology of nondiabetic focal segmental glomerulosclerosis

**DOI:** 10.1038/s41598-023-49861-0

**Published:** 2023-12-19

**Authors:** Guilherme Lopes-Gonçalves, Juliana Martins Costa-Pessoa, Ruan Pimenta, Ana Flavia Tostes, Eloisa Martins da Silva, Felipe Lourenço Ledesma, Denise Maria Avancini Costa Malheiros, Roberto Zatz, Karina Thieme, Niels Olsen Saraiva Câmara, Maria Oliveira-Souza

**Affiliations:** 1 Laboratory of Renal Physiology, Department of Physiology and Biophysics, Institute of Biomedical Sciences, University of Sao Paulo, 1524 Prof. Lineu Prestes Avenue, Sao Paulo, 05508-000 Brazil; 2 Laboratory of Medical Investigation (LIM 55), Urology Department, Faculty of Medicine, University of Sao Paulo, Sao Paulo, Brazil; 3 Laboratory of Neurobiology, Department of Physiology and Biophysics, Institute of Biomedical Sciences, University of Sao Paulo, Sao Paulo, Brazil; 4 Department of Nephrology, Paulista School of Medicine, Federal University of Sao Paulo, Sao Paulo, Brazil; 5 Department of Pathology, Faculty of Medicine, University of Sao Paulo, Sao Paulo, Brazil; 6 Renal Division, Department of Clinical Medicine, Faculty of Medicine, University of Sao Paulo, Sao Paulo, Brazil; 7 Laboratory of Cellular and Molecular Bases of Renal Physiology, Department of Physiology and Biophysics, Institute of Biomedical Sciences, University of Sao Paulo, Sao Paulo, Brazil; 8 Laboratory of Transplantation Immunobiology, Department of Immunology and Biophysics, Institute of Biomedical Sciences, University of Sao Paulo, Sao Paulo, Brazil

**Keywords:** Kidney, Nephrons, Glomerulus

## Abstract

Focal segmental glomerulosclerosis (FSGS) is the leading cause of nephrotic syndrome, which is characterized by podocyte injury. Given that the pathophysiology of nondiabetic glomerulosclerosis is poorly understood and targeted therapies to prevent glomerular disease are lacking, we decided to investigate the tight junction protein claudin-1 and the histone deacetylase sirtuin-1 (SIRT1), which are known to be involved in podocyte injury. For this purpose, we first examined SIRT1, claudin-1 and podocin expression in kidney biopsies from patients diagnosed with nondiabetic FSGS and found that upregulation of glomerular claudin-1 accompanies a significant reduction in glomerular SIRT1 and podocin levels. From this, we investigated whether a small molecule activator of SIRT1, SRT1720, could delay the onset of FSGS in an animal model of adriamycin (ADR)-induced nephropathy; 14 days of treatment with SRT1720 attenuated glomerulosclerosis progression and albuminuria, prevented transcription factor Wilms tumor 1 (WT1) downregulation and increased glomerular claudin-1 in the ADR + SRT1720 group. Thus, we evaluated the effect of ADR and/or SRT1720 in cultured mouse podocytes. The results showed that ADR [1 µM] triggered an increase in claudin-1 expression after 30 min, and this effect was attenuated by pretreatment of podocytes with SRT1720 [5 µM]. ADR [1 µM] also led to changes in the localization of SIRT1 and claudin-1 in these cells, which could be associated with podocyte injury. Although the use of specific agonists such as SRT1720 presents some benefits in glomerular function, their underlying mechanisms still need to be further explored for therapeutic use. Taken together, our data indicate that SIRT1 and claudin-1 are relevant for the pathophysiology of nondiabetic FSGS.

## Introduction

Focal segmental glomerulosclerosis (FSGS) is the leading cause of nephrotic syndrome^1–4^. By histology, FSGS is characterized by a scarring pattern observed in progressive kidney disease, which involves the obliteration of segmental areas of the glomerular tuft by increased matrix deposition^5–7^. The clinical presentation and prognosis of FSGS differ according to the FSGS variant, so an accurate diagnosis is essential for management^8^. Despite some limitations of use and potential side effects, glucocorticoids and calcineurin inhibitors are the basis for treating FSGS disorders^9^. Therefore, the search for possible therapies that can minimize podocyte injury and proteinuria and delay the progression of chronic kidney disease (CKD) is a global challenge.

Primary FSGS is linked to circulating factors that affect podocyte structure^10,11^. The most important point of cell injury in primary FSGS is the podocyte since it is considered a podocytopathy^4,12^. The normal podocyte slit diaphragm maintains the glomerular ultrafiltration barrier structure and acts to prevent the filtration of large amounts of protein^13,14^. In the context of FSGS, these cells are subjected to flattening, oxidative stress, endoplasmic reticulum stress, dedifferentiation, detachment, and apoptosis^13–18^. All these events provide favorable conditions for its evolution, resulting in end-stage CKD, making patients dependent on dialysis or kidney transplantation.

Claudins are tight junction membrane proteins that regulate the paracellular transport of electrolytes in the kidney epithelium, cell polarity, and differentiation^19,20^. Claudin-1 is expressed in the parietal epithelial cells (PECs) of the Bowman capsule, distal tubule, and collecting duct^21^. Glomerular claudin-1 has been reported as a marker of early FSGS^22,23^. To date, in podocyte-specific claudin-1 transgenic mice, the overexpression of claudin-1 interacts with the nephrin/podocin arrangement, affects slit diaphragm structure, and ultimately leads to albuminuria^24,25^. Previous studies reported reduced renal SIRT1 protein expression, especially in a model of diabetes, and these studies have examined the effect of proximal tubular SIRT1 and epigenetic regulation of claudin-1 in podocytes^24,26,27^ on diabetic kidney disease, but this interplay is still unclear in the context of nondiabetic FSGS, since tubular structures are not primarily affected in this condition.

Sirtuins are deacetylases dependent on nicotinamide adenine dinucleotide (NAD^+^), which interact with histones, promoting post-translational modifications that result in chromatin silencing and suppression of gene transcription, thus regulating a wide range of physiological functions^28^. Sirtuin-1 (SIRT1) is a well-investigated isoform in the kidney that is expressed in podocytes, tubular cells, and interstitial cells^29,30^. Thus far, renal SIRT1 inhibits oxidative stress^31^, inflammation^32^, fibrosis^33,34^, and cell apoptosis^35,36^.

Given that SIRT1 can be pharmacologically stimulated both in vivo and in vitro using synthetic agonists such as SRT1720^37,38^, we hypothesize that this compound could be useful in preventing glomerular injury. Thus, we sought to investigate the relationship between SIRT1 and claudin 1 in human kidney biopsies, in an experimental adriamycin (ADR)-induced nephropathy model, and in immortalized mouse podocytes.

## Results

### Glomerular expression of SIRT1 and claudin-1 in human FSGS renal biopsies

**Figure 1:**
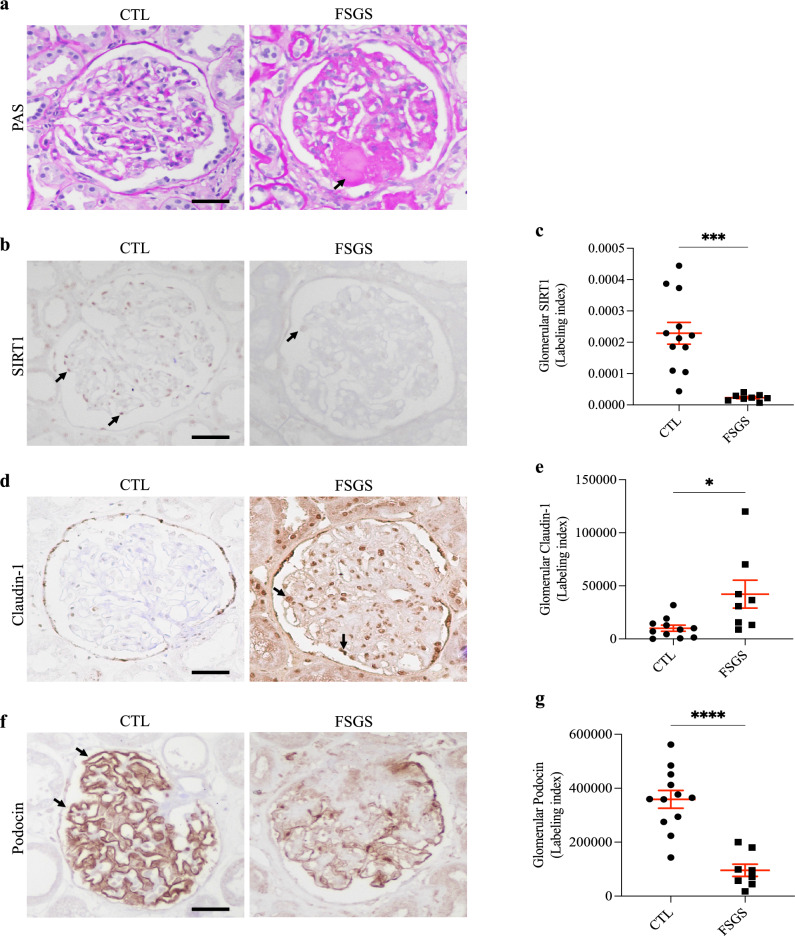
In focal segmental glomerulosclerosis, the glomerular loss of sirtuin-1 was accompanied by claudin-1 upregulation and podocyte injury. (**a**) Representative periodic acid-Schiff (PAS) staining of kidney sections (4 µm thick) from CTL or FSGS patients. The black arrows indicate positive staining for segmental glomerulosclerosis. (**b**) Representative immunohistochemistry micrographs (4 µm thick) and graphic presentation (**c**) show that nuclear SIRT1 was significantly reduced in FSGS patients. The black arrows indicate positive staining for nuclear SIRT1. (**d**) Representative immunohistochemistry micrographs (4 µm thick) and graphic presentation (**e**) show the upregulation of glomerular claudin-1 in FSGS patients. The black arrows indicate positive staining for glomerular claudin-1. (**f**) Representative immunohistochemistry micrographs (4 µm thick) and graphic presentation (**g**) show that glomerular podocin staining was greatly reduced in FSGS patients. The black arrows indicate positive staining for glomerular podocin. Statistical analyses were performed by parametric unpaired *t* test with Welch’s correction. Values are expressed as the mean ± SEM. *P < 0.05, ***P < 0.001, ****P < 0.0001. Scale bar, 20 µm. *CTL* control patients (graph circles), *FSGS* patients diagnosed with nondiabetic focal segmental glomerulosclerosis (graph squares), *SIRT1* sirtuin-1.

Initially, human glomerulosclerosis was confirmed by histological PAS staining. FSGS biopsies showed a typical glomerular scarring pattern on light microscopy (Fig. 1a). Through immunohistochemistry and specific antibodies, glomerular SIRT1, claudin-1, and podocin labeling were evaluated. Our results showed a significant reduction in glomerular nuclear SIRT1 labeling in FSGS patients compared to CTL patients (Fig. 1b,c). However, a significant increase in the glomerular claudin-1 labeling index could be observed in FSGS patients compared to CTL patients (Fig. 1d,e). Similar to SIRT1, podocin staining was greatly reduced in FSGS patients compared to CTL patients (Fig. 1f,g).

### Chronic SRT1720 administration was not effective in suppressing glomerular claudin-1 expression; however, it maintained podocyte differentiation status

**Figure 2:**
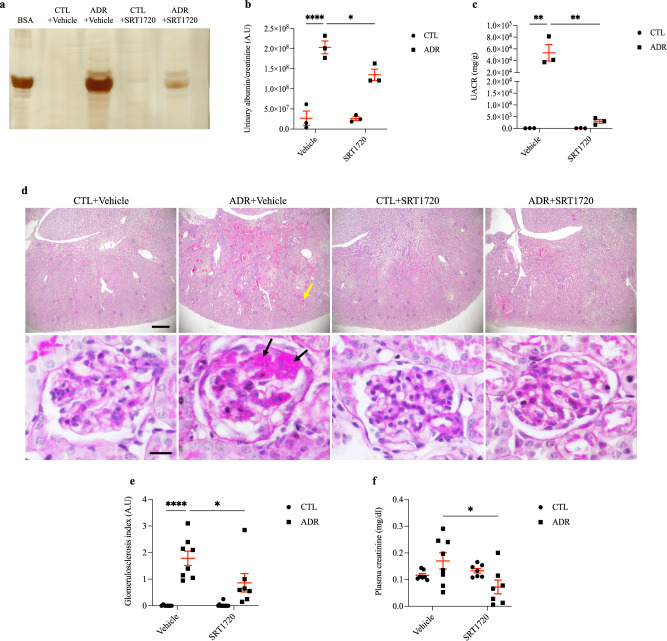
Chronic SRT1720 administration alleviates albuminuria and glomerulosclerosis in mice with adriamycin-induced nephropathy. (**a**) The 10% SDS-PAGE analysis and graphic presentation (**b**) show the abundance of urinary albumin, which was markedly reduced by SRT1720 chronic administration. Statistical analysis was performed by two-way ANOVA. A significant interaction between ADR/SRT1720 was observed by two-way ANOVA (F = 5.76; DF = 1; *P* = 0.043; Table 1). A bovine serum albumin standard (BSA, 1 mg/ml) was loaded on the adjacent lane (left). The cropped gel is displayed, and the full-length gel is included in the supplementary files. (**c**) Graphic presentation of the urinary albumin-to-creatinine ratio (mg/g). Statistical analysis was performed by two-way ANOVA. A significant interaction between ADR/SRT1720 was observed by two-way ANOVA (F = 12.8; DF = 1; *P* = 0.0072; Table 1). (**d**) Representative PAS micrographs (4 µm thick) and graphic presentation (**e**) show that chronic SRT1720 administration attenuated ADR + vehicle-induced glomerular sclerotic lesions, as well as protein cast deposition inside tubular structures. The yellow arrow shows protein casts inside tubules, and black arrows indicate positive staining for segmental glomerulosclerosis. Statistical analysis was performed by two-way ANOVA. A significant interaction between ADR/SRT1720 was observed by two-way ANOVA (F = 5.56; DF = 1; *P* = 0.025; Table 1). Scale bar, 100 µm (40 ×, kidney cortex) and 10 µm (400 ×, glomerular section). (**f**) Plasma creatinine analysis. Statistical analysis was performed by two-way ANOVA. A significant interaction between ADR/SRT1720 was observed by two-way ANOVA (F = 6.93; DF = 1; *P* = 0.014; Table 1). Values are expressed as the mean ± SEM. *P < 0.05, **P < 0.01, ****P < 0.0001. *CTL* control mice (graph circles), *ADR* adriamycin-injected mice (graph squares), *SRT1720* specific SIRT1 agonist, *SDS-PAGE* sodium dodecyl sulfate-polyacrylamide gel electrophoresis, *A.U* arbitrary units, *UACR* urinary albumin-to-creatinine ratio, *ANOVA* analysis of variance, *F* F-statistic, *DF* degrees of freedom.

**Figure 3:**
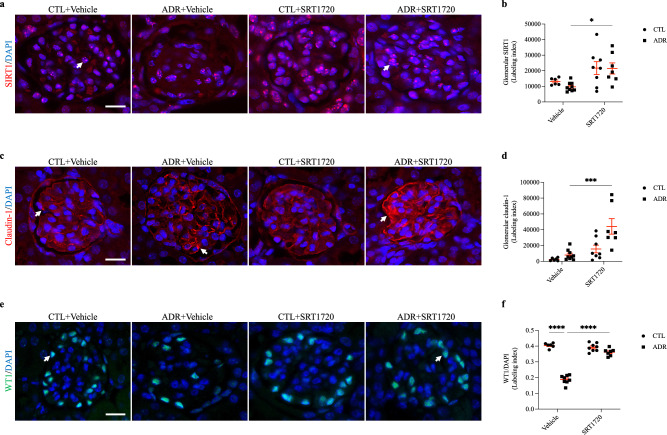
Chronic SRT1720 administration fails to attenuate glomerular claudin-1 in mice with adriamycin-induced nephropathy, although WT1 levels are maintained. (**a**) Representative micrographs (4 µm thick) and graphic presentation (**b**) confirming the upregulation of SIRT1 (red) by chronic SRT1720 administration. The white arrows indicate positive staining for nuclear SIRT1. Statistical analysis was performed by two-way ANOVA. (**c**) Representative micrographs (4 µm thick) and graphic presentation (**d**) show that chronic SRT1720 administration increases glomerular claudin-1 expression in ADR + vehicle-treated mice. The white arrows indicate positive staining for glomerular claudin-1. Statistical analysis was performed by two-way ANOVA. A significant interaction between ADR/SRT1720 was observed by two-way ANOVA (F = 4.37; DF = 1; *P* = 0.046; Table 1). (**e**) Representative micrographs (4 µm thick) and graphic presentation (**f**) show that chronic SRT1720 administration attenuates WT1 depletion in ADR + SRT1720-treated mice. The white arrows indicate positive staining for WT1. Statistical analysis was performed by two-way ANOVA. A significant interaction between ADR/SRT1720 was observed by two-way ANOVA (F = 108; DF = 1; *P* < 0.0001; Table 1). Scale bar, 10 µm. Values are expressed as the mean ± SEM. *P < 0.05, ***P < 0.001, ****P < 0.0001. *CTL* control mice (graph circles), *ADR* adriamycin-injected mice (graph squares), *SIRT1* sirtuin-1, *SRT1720* specific SIRT1 agonist, *WT1* Wilms tumor 1, *ANOVA* analysis of variance, *F* F-statistic, *DF* degrees of freedom.

For determination of the role of SIRT1 in FSGS development, BALB/c mice received a single ADR injection (10 mg/kg) and prolonged administration of SRT1720 (20 mg/kg) or vehicle (10% DMSO) for 14 days. After this period, ADR + vehicle-treated mice showed a marked increase in urinary albumin excretion, which could be significantly prevented by SRT1720 administration (Fig. 2a–c, Supplementary Fig. S1 and Table 1). Tubular protein casts and advanced glomerulosclerosis were also observed with PAS staining in the ADR + vehicle group (Fig. 2d). These results confirm the experimental model induction. In contrast, specific SIRT1 stimulation in the ADR + SRT1720 group attenuated the glomerulosclerosis index, tubular damage, and tubular protein cast formation and reduced kidney damage (Fig. 2d,e, and Table 1). Although in the ADR + vehicle group, plasma creatinine levels did not significantly increase compared to those in the CTL + vehicle group, SRT1720 administration was able to reduce this parameter (Fig. 2f and Table 1). The ADR + vehicle-treated mice also exhibited a significant drop in weight gain, which was not prevented by SRT1720 administration (Supplementary Fig. S1 and Table 1). Furthermore, no significant changes regarding water intake, food ingestion, kidney weight, or creatinine clearance were observed (Supplementary Fig. S1 and Table 1). SIRT1, claudin-1, and the transcription factor Wilms tumor 1 (WT1) were assessed by immunofluorescence. Treatment with ADR + vehicle resulted in a slight and nonsignificant decrease in SIRT1 expression compared to that in the CTL + vehicle group. However, an increased number of SIRT1-positive cell nuclei was observed in glomerular cross-sections from the ADR + SRT1720 group compared to the ADR + vehicle group (Fig. 3a,b, and Table 1). ADR + vehicle treatment induced only a slight increase in glomerular claudin-1 expression compared to that of the CTL + vehicle group, while chronic SRT1720 administration was unable to attenuate glomerular claudin-1 expression and significantly increased protein labeling in the ADR + SRT1720 group compared to the ADR + vehicle group (Fig. 3c,d and Table 1). Next, we evaluated the podocyte number by glomerular WT1 labeling. Our data demonstrated a significant loss of WT1 in the ADR + vehicle-treated animals compared to the CTL + vehicle animals, which was clearly prevented by the SIRT1-specific agonist in the ADR + SRT1720 group (Fig. 3e,f and Table 1). To complement our data and to confirm the effects of SRT1720 on kidney function, we treated a group of animals with vehicle (10% DMSO). In this analysis, kidney morphology was unaffected, and there were no significant differences in creatinine or plasma urea values compared to those of the CTL group (Supplementary Fig. S2 and Supplementary Table S1). In summary, these data indicate that despite claudin-1 upregulation in the ADR + SRT1720 group, SIRT1 chronic stimulation can play a protective role against FSGS podocyte injury. To investigate this possibility, we used mouse podocyte culture.

**Table 1:** Statistical analysis of animal experimentation data.

Parameters	ADR + vehicle			SRT1720 + vehicle			Interaction		
	F value	DF value	*P* value	F value	DF value	*P* value	F value	DF value	*P* value
Urinary albumin/urinary creatinine, A.U	103	1	< 0.0001	6.06	1	0.0392	5.76	1	0.0432
Urinary albumin-to-creatinine ratio, mg/g	15.9	1	0.0040	12.8	1	0.0072	12.8	1	0.0072
Glomerulosclerosis index	42.8	1	< 0.0001	5.10	1	0.0317	5.56	1	0.0253
Plasma creatinine, mg/dl	0.0268	1	0.8713	3.43	1	0.0759	6.93	1	0.0143
Weight gain, g	17.8	1	0.0003	0.231	1	0.6351	1.03	1	0.3202
Food intake, g/day	0.996	1	0.3278	2.61	1	0.1189	0.174	1	0.6800
Water intake, ml/day	6.684	1	0.0160	0.1790	1	0.6759	1.169	1	0.2900
Kidney weight/final body weight, mg/g	10.4	1	0.0035	2.76	1	0.1093	0.117	1	0.7351
Creatinine clearance, µl/min	9.69	1	0.0045	20.2	1	0.0001	2.75	1	0.1096
Glomerular SIRT1, labeling index	0.406	1	0.5294	12.4	1	0.0016	0.305	1	0.5853
Glomerular claudin-1, labeling index	9.69	1	0.0045	20.2	1	0.0001	4.37	1	0.0464
WT1/DAPI, labeling index	192	1	< 0.0001	81.4	1	< 0.0001	108	1	< 0.0001

Two-way ANOVA was performed to compare data. The F, DF and exact *P* values considering ADR, SRT1720 and interaction are described. Significance was assumed when *P* < 0.05. *ADR* adriamycin, *SRT1720* specific SIRT1 agonist, *ANOVA* analysis of variance, *F* F-statistic, *DF* degrees of freedom, *A.U* arbitrary units.

### Claudin-1 protein expression increases in short-term ADR-treated mouse podocytes and is counteracted by acute stimulation of SIRT1

**Figure 4:**
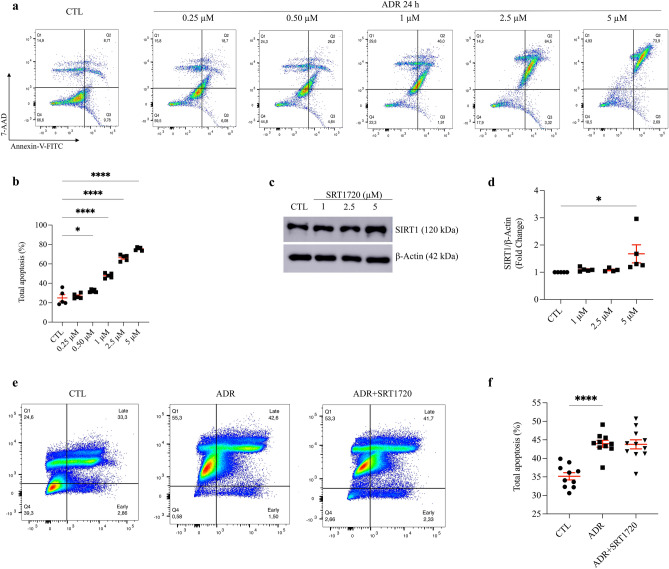
Prior stimulation of sirtuin-1 with SRT1720 does not attenuate podocyte adriamycin-induced apoptosis in vitro. (**a**) Representative flow cytometry analysis and graphic presentation (**b**) show that ADR increases podocyte apoptosis in a dose-dependent manner. Cells were treated with various concentrations of ADR for 24 h, as indicated. Q1, cells in necrosis; Q2, cells in late apoptosis; Q3, cells in early apoptosis; and Q4, healthy cells. Total apoptosis means the sum of early and late apoptosis. Statistical analysis was performed by one-way ANOVA. (**c**) Representative immunoblots (10% SDS-PAGE) and graphic presentation (**d**) show SIRT1 protein abundance in SRT1720-treated cells, as indicated. The cropped blot is displayed, and the full-length gel is included in the supplementary files. (**e**) Representative flow cytometry analysis and graphic presentation (**f**) show that SRT1720 did not attenuate ADR-induced podocyte apoptosis. ADR cells were treated with 1 µM ADR for 24 h. ADR + SRT1720 cells were pretreated with SRT1720 [5 µM] for 24 h and then treated with ADR [1 µM] for 24 h. Total apoptosis means the sum of early and late apoptosis. Statistical analysis was performed by one-way ANOVA. Values are expressed as the mean ± SEM. *P < 0.05, ****P < 0.0001. *CTL* control cells, *ADR* adriamycin-treated cells, *SIRT1* sirtuin-1, *SRT1720* specific SIRT1 agonist, *SDS-PAGE* sodium dodecyl sulfate-polyacrylamide gel electrophoresis, *ANOVA* analysis of variance.

To investigate the interplay between SIRT1 and claudin-1, we examined the ADR dose-dependent effect on podocyte apoptosis in vitro. First, podocytes were treated at concentrations of 0.25 µM, 0.50 µM, 1 µM, 2.5 µM, and 5 µM for 24 h in RPMI 1640 culture medium with 5% FBS. Then, apoptosis was analyzed by flow cytometry. In the present study, Annexin-V-FITC and 7-AAD markers were used. Next, incubation of podocytes with ADR at concentrations of 1 µM, 2.5 µM, and 5 µM led to a significant increase in the total apoptosis rate compared to that of the CTL group (Fig. 4a,b). Therefore, the concentration used for subsequent experiments was 1 µM.

Likewise, for SRT1720 standardization, cells were treated at concentrations of 1 µM, 2.5 µM, and 5 µM for 24 h in the same basal media. Subsequently, SIRT1 protein expression was evaluated by immunoblotting. SRT1720 treatment at 5 µM resulted in a significant increase in SIRT1 protein expression after 24 h compared to that of the CTL group. However, SRT1720 at 1 µM or 2.5 µM did not induce significant changes in SIRT1 expression relative to that of the CTL group (Fig. 4c,d and Supplementary Fig. S3). Therefore, the selected concentration of SRT1720 was 5 µM. Additionally, in apoptosis experiments, the results showed that prior stimulation of SIRT1 with SRT1720 was unable to attenuate ADR-induced apoptosis in podocytes in vitro (Fig. 4e,f).

**Figure 5:**
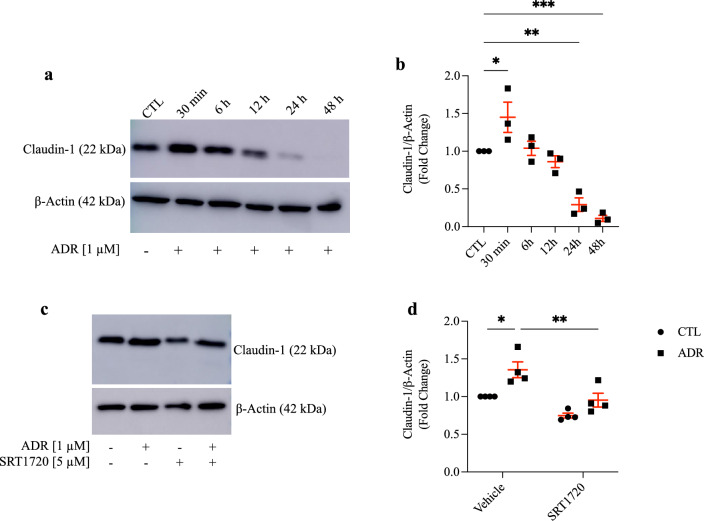
Adriamycin-induced podocyte apoptosis is preceded by a rapid increase in claudin-1 expression, which is counteracted by sirtuin-1 acute stimulation with SRT1720. (**a**) Representative immunoblots (15% SDS-PAGE) and graphic presentation (**b**) show claudin-1 protein abundance in ADR [1 µM]-treated cells at various time points, as indicated. The cropped blot is displayed, and the full-length gel is included in the supplementary files. Statistical analysis was performed by one-way ANOVA. (**c**) Representative immunoblots (15% SDS-PAGE) and graphic presentation (**d**) show claudin-1 protein abundance in SRT1720 [5 µM, 24 h] + ADR [1 µM, 30 min] treated cells, as indicated. The cropped blot is displayed, and the full-length gel is included in the supplementary files. Statistical analysis was performed by two-way ANOVA. Values are expressed as the mean ± SEM. *P < 0.05, **P < 0.01, ***P < 0.001. *CTL* control cells (graph circles), *ADR* adriamycin-treated cells (graph squares), *SIRT1* sirtuin-1, *SRT1720* SIRT1-specific agonist, *SDS-PAGE* sodium dodecyl sulfate-polyacrylamide gel electrophoresis, *ANOVA* analysis of variance.

Claudin-1 protein expression was evaluated within the time-response curve of 30 min, 6 h, 12 h, 24 h, and 48 h based on 1 µM ADR treatment. Our results showed a significant increase in claudin-1 protein expression at the 30 min time point compared to that of the CTL group, as well as a significant decrease after 24 h and 48 h of treatment (Fig. 5a,b, and Supplementary Fig. S3). Given that the highest claudin-1 expression occurred 30 min after ADR incubation, we decided to explore the SIRT1/claudin-1 crosstalk at this experimental time point. For this purpose, podocytes were pretreated with 5 µM SRT1720 for 24 h and then treated with 1 µM ADR for 30 min. Our results demonstrated that early and acute SIRT1 stimulation markedly reduced ADR-induced claudin-1 expression (Fig. 5c,d, Supplementary Fig. S3, and Table 2).

**Table 2:** Statistical analysis of cell culture data.

Parameter	ADR			SRT1720			Interaction		
	F value	DF value	*P* value	F value	DF value	*P* value	F value	DF value	*P* value
Claudin-1/β-actin, fold change	15.4	1	0.0020	21.2	1	0.0006	1.13	1	0.3087

Two-way ANOVA was performed to compare data. The F, DF and exact *P* values considering ADR, SRT1720 and interaction are described. Significance was assumed when *P* < 0.05. *ADR* adriamycin, *SRT1720* specific SIRT1 agonist, *ANOVA* analysis of variance, *F* F-statistic, *DF* degrees of freedom.

### Cellular distribution of SIRT1 and claudin-1 is changed in podocytes treated with ADR

**Figure 6:**
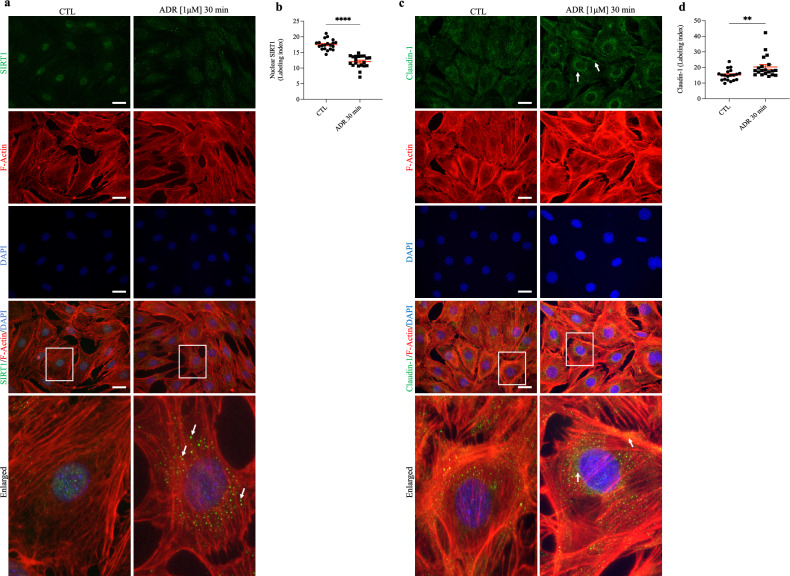
Adriamycin treatment leads to podocyte sirtuin-1 translocation from the nucleus to the cytoplasm, as well claudin-1 upregulation. (**a**) Representative micrographs and graphic presentation (**b**) show the nuclear output of SIRT1 (green) in ADR [1 µM, 30 min]-treated cells. White arrows indicate cytoplasmic SIRT1. (**c**) Representative micrographs and graphic presentation (**d**) show increased claudin-1 staining in ADR [1 µM, 30 min]-treated cells. White arrows indicate concentrated claudin-1 (green) spots on cell membranes, as well as the perinuclear region. Values are expressed as the mean ± SEM. **P < 0.01, ****P < 0.0001. Statistical analyses were performed by unpaired *t* test with Welch’s correction. *CTL* control cells (graph circles), *ADR* adriamycin-treated cells (graph squares), *SIRT1* sirtuin-1.

To assess cellular SIRT1 and claudin-1 dynamics in mouse podocytes, we treated cells with 1 µM ADR for 30 min. Our immunofluorescence studies showed that nuclear SIRT1 expression was sharply reduced in the ADR-treated cells (Fig. 6a,b), spreading in the cytoplasm, while claudin-1 staining increased significantly, especially in the perinuclear region and plasma membrane (Fig. 6c,d).

## Discussion

Interestingly, our study reveals that in nondiabetic human FSGS, reduced SIRT1 expression was associated with increased claudin-1 expression and a decrease in podocin protein expression and organization, which suggests communication between these proteins in FSGS-induced podocyte injury.

ADR-induced nephropathy results in glomerular injury in rodents, which is similar to human glomerular disease^39,40^. This model has been widely studied and allows elucidation of the mechanisms involved in CKD progression; thus, it is suitable for testing interventions that could attenuate kidney and podocyte injury^40^. For this purpose, we used SRT1720, a selective SIRT1 agonist. Regarding ADR + vehicle-treated mice, although the increase in plasma creatinine levels was not significant, strong urinary albumin excretion, low expression of WT1, and a high degree of glomerulosclerosis were clearly prevented by SRT1720 chronic administration, suggesting that SIRT1 contributes to attenuating the progression of glomerular injury in the experimental model of ADR-induced FSGS. The transcription factor WT1 is essential to stimulate nephrin, podocin, and podocalyxin gene expression, which are needed to maintain podocyte function and differentiation status; therefore, ADR-induced loss of WT1 suggests dedifferentiation, cytoskeletal disruption and podocyte detachment^41^. Furthermore, in an analysis of the ADR + vehicle group, although creatinine clearance did not decrease compared to that of the CTL + vehicle group, the results were consistent with those of Wang et al., who observed a decrease in glomerular function approximately 28 days after ADR administration in BALB/c mice^42^. SRT1720 administration did not reverse the body weight loss induced by ADR + vehicle. Notably, the ADR-induced nephropathy induces many metabolic alterations^43^, which could impact the ability to recover normal weight even with a SIRT1 activator.

In the glomeruli of ADR + vehicle-treated mice, claudin-1 was expressed in a manner compatible with the podocyte foot process, although the increase was not significant compared to that in the CTL + vehicle group. Claudin-1 is a tight junction protein that plays a critical role in regulating PECs and endothelial cell function^44,45^. Although the overall function of claudin-1 is conserved across species, there are differences in its expression levels and distribution pattern, as shown in humans and mice. These discrepancies could be due to evolutionary divergence or variations in tissue-specific regulation. The literature has shown that many proteins can facilitate the shifting of claudin-1 localization, such as protein kinase A (PKA) and protein kinase C (PKC)^46^, or soluble factors, such as interleukin-1 beta (IL-1β)^47,48^, transforming growth factor beta-1 (TGF-β1)^49^ and tumor necrosis factor alpha (TNF-α)^50^. Using a transgenic mouse model, Gong et al. overexpressed claudin-1 specifically in podocytes. After 4 weeks, there was significant albuminuria compared to the control. The authors also observed that the increase in claudin-1 expression was compatible with podocyte ultrastructural changes, decreasing the expression of slit diaphragm components such as nephrin and podocin^51^. Taken together, our results corroborate these findings.

Curiously, in ADR + SRT1720-treated mice, SRT1720 administration increased glomerular claudin-1 labeling. The interplay between SIRT1 and claudin-1 was elegantly described by Hasegawa et al. using diabetic mouse models^24^. Under diabetic conditions, the decreased levels of proximal tubular nicotinamide mononucleotide and podocyte SIRT1 indicate H3 and H4 histone acetylation and diminished H3K9 methylation. These histone modifications decrease the activity of DNA (cytosine-5)-methyltransferase 1 (Dnmt1), which in turn causes hypomethylation of the claudin-1 gene, leading to increased podocyte expression of claudin-1^25,26,28^. Indeed, in several experimental models, including diabetic nephropathy and renal fibrosis induced by unilateral ureteral obstruction (UUO), SIRT1 is described as a therapeutic target, reducing endoplasmic reticulum stress and fibrosis^52^. In contrast, Ponnusamy et al. observed that in a UUO model, the SIRT1 agonist SRT1720 accelerated the deposition of collagen fibers and increased the expression of fibroblast activation markers such as alpha smooth muscle actin (α-SMA), collagen I and fibronectin^53^. The fibrogenic effect of SRT1720 was associated with connections between SIRT1 and other proteins, such as increased phosphorylation of epidermal growth factor receptor (EGFR Tyr1068) and platelet-derived growth factor β (PDGFRβ Tyr751). SRT1720 also increased the phosphorylation of STAT3 (Tyr705) and AKT (Ser473) in renal fibroblasts^53^ and PKA (Thr197) in vascular smooth muscle cells^54^. Additionally, PKA phosphorylation is known to regulate the subcellular expression of claudin-1 in myeloma cells^46^. In this sense, we strongly believe that in our experimental model, PKA was targeted by SRT1720, resulting in increased claudin-1 expression. In this sense, despite their short-term benefits for kidney function, caution should be taken when using SIRT1 activators to avoid late podocyte injury.

Given that ADR induces podocyte injury, we investigated the effect of ADR on cell apoptosis. First, we standardized ADR concentrations to validate podocyte injury. Our results showed that apoptosis was dependent on the ADR dose, as observed by Yi et al.^55^. In the context of podocytes, apoptosis is essential for glomerulosclerosis progression^56^. The ADR effects on podocytes have already been described by previous studies: generation of reactive oxygen species (ROS), increased apoptosis rate, expression of cleaved caspase-3 and BAX, and reduced Bcl-2^57^. A recent study reported that an ADR concentration of 1 µM on podocytes for 48 h promoted chromatin condensation and nuclear fragmentation^58^. It has been observed that a concentration of 2 µg/ml (equivalent to 3.44 µM) induces epithelial-to-mesenchymal transition (EMT), mainly by increasing integrin-linked kinase activity^59^. Furthermore, a concentration of 3 µg/ml (equivalent to 5.17 µM) for 72 h was associated with podocyte advanced glycation end products and ROS synthesis^60^. In contrast, in our experimental model, concentrations above 5 µM would be impractical, as the rate of apoptosis would be up to 80%, characterizing nonviable cells.

To date, few studies have investigated SRT1720 in podocytes in vitro. Wang et al. evaluated the inhibitory effect of astragaloside IV on podocyte EMT induced by high glucose concentrations^61^. In this case, podocytes were treated with SRT1720 at a concentration of 10 µM for 48 h to verify the role of SIRT1. To enhance proximal tubular SIRT1, studies have shown that the SRT1720 concentration varies from 2.5 to 10 µM^62–64^. Thus, we decided to standardize SRT1720 concentration in podocytes to evaluate SIRT1 protein expression. According to our data, SRT1720 at a concentration of 5 µM increased SIRT1 protein expression in podocytes after a 24 h treatment period. Based on the ADR and SRT1720 concentrations in in vitro studies, we observed a substantial increase in claudin-1 expression after 30 min of podocyte treatment with 1 µM ADR. The initial increase in claudin-1 expression at 30 min may be a response to the cellular damage caused by ADR. In this case, podocytes could try to restructure the cytoskeleton and their tight junctions by increasing claudin-1. After an initial response, cells enter a more severe state of stress or injury, which can result in a rapid decrease in claudin-1 expression. This process can be related to cellular mechanisms involved in apoptosis.

Since chronic and concomitant administration of SRT1720 did not attenuate claudin-1 expression in the glomeruli of ADR + SRT1720-treated mice, we decided to examine whether prior stimulation of SIRT1 with SRT1720 could affect claudin-1 expression in podocyte culture. Our results showed that prior stimulation of SIRT1 in podocytes prevented ADR-induced claudin-1 protein expression. Our study reveals that podocytes produce their own SIRT1, which enhances their local regulatory responses regardless of proximal tubule/podocyte crosstalk, as observed in diabetic models. Next, we observed that the treatment of podocytes with 1 µM ADR for 30 min resulted in SIRT1 translocation from the nucleus to the cytoplasm, as well as recruitment of claudin-1 to the perinuclear/plasma membrane. These modifications in the expression pattern of SIRT1 and claudin-1 could be linked to cellular adaptation and actin cytoskeleton changes, respectively, which were induced by treatment with ADR^65,66^.

Taken together, our data suggest that the lack of SIRT1 is associated with increased glomerular claudin-1 and podocyte injury in human FSGS. In an animal model of ADR-induced nephropathy, the chronic administration of SRT1720 prevented renal function changes induced by ADR. In immortalized mouse podocytes, although SRT1720 did not attenuate ADR-induced apoptosis, the agonist decreased claudin-1 protein expression. This finding suggests that in all three circumstances, SIRT1 can regulate the activity and expression of podocyte claudin-1. On the other hand, attention should be given when using SIRT1 agonists as therapeutic targets for glomerular diseases since they can stimulate renal fibrogenesis.

## Methods

### Human biopsies

All methods were performed in accordance with the relevant guidelines, regulations, and the Declaration of Helsinki. Kidney biopsy samples were obtained from Hospital das Clínicas FMUSP, University of Sao Paulo, with written informed consent. Twelve control patients (CTL; autopsied or healthy kidney parenchyma removed due to renal carcinoma) and eight patients diagnosed with FSGS were selected. Patients diagnosed with diabetic glomerulosclerosis were excluded from the study. The baseline characteristics of the FSGS patients are described in Supplementary Table S2. The protocol was approved by the Research Ethics Committee (CEP/ICB-USP), Institute of Biomedical Sciences, University of Sao Paulo, Brazil, protocol no. 3.812.162.

### Adriamycin nephropathy

All methods were performed in accordance with the relevant guidelines, regulations and with ARRIVE (Animal Research: Reporting of In Vivo Experiments) guidelines. Male BALB/c mice aged 4 weeks were acquired from the animal care facility of the Medicine School, University of Sao Paulo, and kept in the experimental animal care facility of the Department of Physiology and Biophysics, Institute of Biomedical Sciences, University of Sao Paulo, Brazil. All animals were maintained under standard conditions (22 °C, 12/12-h light/dark cycles, 60% relative humidity, standard mice chow-fed, and ad libitum water) for 1 week. Experiments began when the animals reached 5 weeks of age. Adriamycin hydrochloride and SRT1720 were purchased from Cayman Chemicals (Cayman Chemicals, Ann Arbor, MI, USA). ADR-induced nephropathy was induced by a single slow intravenous administration of ADR (10 mg/kg body weight, diluted in 0.9% NaCl) into the tail vein. SRT1720 was first dissolved in 100% DMSO and then diluted in 10% DMSO with 0.9% NaCl. SRT1720 was administered intraperitoneally daily for 14 days (20 mg/kg body weight). Mice were randomly distributed into four groups: control + vehicle group (CTL + vehicle), ADR + vehicle nephropathy group, CTL + SRT1720 group, and ADR + SRT1720 group. The groups without ADR received the same dose of 0.9% NaCl via the tail vein. The groups without SRT1720 received the same dose of vehicle (10% DMSO) intraperitoneally. After this, mice were individually placed inside metabolic cages (Tecniplast, Buguggiate, VA, Italy) for 24 h urine collection. The animals were kept under inhalation anesthesia on the last day using 5% isoflurane. After the complete loss of pain reflexes, an abdominal incision was made using a scalpel (DL Micof, Sao Paulo, SP, BR). Blood samples were collected from the heart, and the kidneys were removed. Euthanasia was performed by exsanguination. All animal procedures were approved by the Ethics Committee on Animal Use of the Institute of Biomedical Sciences, University of Sao Paulo, Brazil (CEUA-ICB/USP), protocol no. 3425170518.

### Kidney function analysis

Plasma and urine creatinine levels were assessed in a Cobas c111 biochemical analyzer (Roche, Indianapolis, IN, USA) according to the manufacturer’s instructions. As previously described^67^, urinary albumin was analyzed by SDS-PAGE after urinary creatinine normalization. Next, gels were stained with a SilverQuest Silver Staining Kit (Invitrogen, Waltham, MA, USA) according to the manufacturer’s instructions. ImageJ software (National Institutes of Health, Bethesda, MD, USA) was used for quantification. In addition, urine albumin was quantified using the Albuwell M 1011 kit (Ethos Bioscience, Logan Township, NJ, USA) according to the manufacturer’s instructions and is presented as the urinary albumin-to-creatinine ratio (UACR).

### Kidney histology

The glomerulosclerosis index (GSI) was assessed in a blinded manner by quantifying at least 20 glomeruli per animal according to a previously described method^68,69^. For each periodic acid-Schiff (PAS) section (4 µm thick), the glomeruli were graded as follows: grade 0, normal; grade 1, sclerotic area of up to 25% (minimal); grade 2, sclerotic area of 26–50% (moderate); grade 3, sclerotic area of 51–75% (moderate to severe); and grade 4, sclerotic area > 75% (severe). The GSI was then calculated using the following formula, where NG is the number of glomeruli with each grade: GSI = [(1 × NG_1_) + (2 × NG_2_) + (3 × NG3) + (4 × NG_4_)]/(NG_0_ + NG_1_ + NG_2_ + NG_3_ + NG_4_).

### Cell culture

As first described by Dr. Peter Mundel^70^, a conditionally immortalized mouse podocyte cell line was certified by Dr. Karlhans Endlich (University of Heidelberg, Germany) and generously provided by Dr. Niels Olsen Saraiva Câmara (Institute of Biomedical Sciences, University of Sao Paulo). Cells were cultured as previously described and summarized here^16,71^. For induction of proliferation, mouse podocytes were grown in 75 cm^2^ flasks coated with type-I collagen and maintained in RPMI 1640 medium (Invitrogen) with 30 IU/ml recombinant mouse interferon γ (BioLegend, San Diego, CA, USA) and 10% fetal bovine serum (FBS, Invitrogen) at a permissive temperature (33 °C). For induction of differentiation, the cells were shifted to 37 °C in RPMI 1640 medium with 5% FBS and without interferon γ for 15 days.

### Flow cytometry

As previously described^16,71^, control and ADR-treated podocytes were trypsinized, and cell staining buffer (BioLegend) was added to the cell samples. The cell suspensions were centrifuged at 2500 rpm for 5 min. This procedure was repeated once more, followed by suspension of the cells in Annexin V binding buffer (BioLegend). A total of 2 × 10^5^ cells were transferred to cytometry sample tubes, and FITC-Annexin V (BioLegend) was incubated for 30 min. After this, 7-AAD (Invitrogen) was added. Cells were subsequently analyzed using a BD FACSCanto II Flow Cytometer/BD LSRFortessa™ X-20 Cell Analyzer (BD Biosciences, Franklin Lakes, NJ, US), calibrated to detect 10,000 or 200,000 events. Cells positive for FITC-Annexin V and 7-AAD were considered apoptotic. The values are shown as percentages (%).

### Immunoblotting

Proteins from control or treated podocytes were extracted using ice-cold 1X RIPA buffer (Abcam, Cambridge, UK) enriched with protease and phosphatase inhibitors (Sigma Aldrich, St. Louis, MO, USA). Immunoblotting was performed on aliquots containing 15 μg/lane of proteins resolved on 8–15% SDS-PAGE as previously described^16^. Protein expression was quantified as the ratio of a specific band to β-actin. The values are represented as the fold change compared to the control group. The primary antibodies used were as follows: anti-claudin-1 (1:2000, #51-9000, Invitrogen), anti-SIRT1 (1:2000, #9475, Cell Signaling Technology, Inc., Danvers, MA, USA), and anti-β-actin (1:10,000, ab6276, Abcam, Cambridge, UK). The following secondary antibodies were used: peroxidase AffiniPure goat anti-rabbit IgG (1:10,000, 111-035-003, Jackson ImmunoResearch Laboratories, Baltimore, MD, USA) and peroxidase AffiniPure goat anti-mouse IgG (1:10,000, 115-035-003, Jackson ImmunoResearch Laboratories).

### Immunofluorescence

Kidney sections (4 µm-thick) were deparaffinized in xylene and rehydrated in a graded ethanol series, ending in running tap water. We performed heat-induced epitope recovery for 5 min using an electric pressure cooker and sodium citrate buffer (10 mM sodium citrate, 0.05% Tween 20, pH 6.0) or Tris–EDTA buffer (10 mM Tris base, 1 mM EDTA solution, 0.05% Tween 20, pH 9.0). After that, the sections were blocked with 1 × TBS plus 1% bovine serum albumin (BSA, VWR, Radnor, PA, USA) for 2 h at room temperature. The blocking solution was removed, and then, the primary antibody was added. The incubation was overnight at 4 °C. The next day, the sections were washed, and *Fab* fragment secondary antibodies were applied to the sections for 1 h at room temperature. Sections were covered with an aqueous mounting medium (Sigma-Aldrich) and coverslips. For cell immunofluorescence, differentiated podocytes were cultured on glass coverslips (Knittel, Braunschweig, NI, DE). Control or treated cells were then fixed with 4% paraformaldehyde (Electron Microscopy Sciences, Hatfield, PA, USA) in 1X PBS (phosphate buffered saline, pH 7.4) for 4 min, permeabilized with 0.1% Triton X-100 in 1 × PBS for 5 min and blocked with 2.5% horse serum solution (Vector Laboratories, Burlingame, CA, USA) for 30 min. Primary antibodies were incubated overnight. Next, the glass coverslips were washed thrice with 1X PBS, followed by secondary antibody incubation for 90 min. F-Actin was labeled with rhodamine phalloidin (Invitrogen). Coverslips were sealed with Vectashield Hardset plus DAPI (Vector Laboratories) for nuclear staining. The fluorescence signal was analyzed with an Eclipse 80i microscope (Nikon, Tokyo, Japan) equipped with a 20 ×/40 × plane objective using excitation wavelengths of 488 nm (green) and 543 nm (red) and NIS-Elements Basic Research software (Nikon). ImageJ software (National Institutes of Health) was used to open files and fluorescence quantification, as described in the Supplementary Methods. The primary antibodies used were as follows: anti-claudin-1 (1:100, #51-9000, Invitrogen), anti-SIRT1 (1:100, #9475, Cell Signaling Technology, Inc.), and anti-WT1 (1:200, ab89901, Abcam). The secondary antibodies used were Alexa Fluor™ 594 AffiniPure Fab Fragment Goat Anti-Rabbit IgG (1:200, 111-587-003, Jackson ImmunoResearch Laboratories), Alexa Fluor™ 488 AffiniPure *Fab* Fragment Goat Anti-Rabbit IgG (1:200, A11070, Jackson ImmunoResearch Laboratories), VectaFluor™ Horse Anti-Rabbit IgG, DyLight™ 488 Antibody kit (ready to use, DI-1788-15, Vector Laboratories), VectaFluor™ Horse Anti-Mouse IgG, and DyLight™ 594 Antibody kit (ready to use, DI-2794-15, Vector Laboratories).

### Immunohistochemistry

Kidney sections (4 µm-thick) were deparaffinized in xylene and rehydrated in a graded ethanol series, ending in running tap water. We performed heat-induced epitope recovery for 5 min using an electric pressure cooker and sodium citrate buffer. After that, endogenous peroxidase was quenched with 3% hydrogen peroxide in 1X PBS for 10 min. Next, the sections were incubated with serum-free protein blocks (Agilent Technologies, Santa Clara, CA, USA) for 2 h at room temperature. The blocking solution was removed, and the primary antibody was applied. The incubation was overnight at 4 °C. The next day, the sections were washed, and a polymer horseradish peroxidase antibody (Agilent Technologies) was applied to the sections for 2 h at room temperature. Subsequent sensitization was performed using 3,3′-diaminobenzidine (Agilent Technologies) and counterstained with methyl green solution (Amresco, Solon, OH, USA). Sections and coverslips were sealed with permanent mounting medium (Fisher Scientific, Hampton, NH, USA). Immunohistochemical staining was analyzed with an Eclipse 80i microscope (Nikon, Tokyo, Japan) equipped with a 20 × plan objective and NIS-Elements Basic Research software (Nikon). ImageJ software (National Institutes of Health) was used to open files and quantification, as described in the Supplementary Methods. The primary antibodies used were as follows: anti-claudin-1 (1:100, #51-9000, Invitrogen), anti-SIRT1 (1:100, #9475, Cell Signaling Technology, Inc.), and anti-podocin (1:400, P0372, Sigma Aldrich). The secondary antibody used was EnVision^+^/Polymer Horseradish Peroxidase Anti-Rabbit Antibody (ready to use, K4003, Agilent Technologies).

### Statistical analysis

Statistical analysis was carried out using GraphPad Prism version 10.1 (GraphPad Software Inc., San Diego, CA, USA). For comparison between two groups, we used a parametric unpaired *t*-test with Welch’s correction. Shapiro–Wilk test was used to consider the normality of data. Comparisons between three or more groups were made using one-way or two-way ANOVA followed by Bonferroni’s and Tukey’s multiple comparisons, respectively. All data were expressed as mean ± standard error of the mean (SEM), whereas *P* < 0.05 was considered significant.

### Statement of ethics

Kidney biopsy samples were obtained from Hospital das Clínicas FMUSP, University of Sao Paulo, with written informed consent. All methods were performed in accordance with the relevant guidelines and the Declaration of Helsinki. The protocol was approved by the Research Ethics Committee (CEP/ICB-USP), Institute of Biomedical Sciences, University of Sao Paulo, Brazil, protocol no. 3.812.162. The animal study was conducted in accordance with ARRIVE (Animal Research: Reporting of In Vivo Experiments) guidelines. All animal procedures were approved by the Institutional Animal Care and Use Committee of the Institute of Biomedical Sciences of the University of Sao Paulo (CEUA-ICB/USP, no. 3425170518).

### Supplementary Information

Supplementary Information.

Supplementary Table S1.

Supplementary Table S2.

## Data Availability

The datasets generated during and/or analyzed during the current study are available from the corresponding author on reasonable request.
