# Supplementary material for: Evaluation of glomerular sirtuin-1 and claudin-1 in the pathophysiology of nondiabetic focal segmental glomerulosclerosis
Source: Sci Rep. 2023 Dec 19;13:22685. doi: 10.1038/s41598-023-49861-0 (PMC10730508; doi:10.1038/s41598-023-49861-0)
Supplement: Supplementary file 1 — Supplementary Information. [file 41598_2023_49861_MOESM1_ESM.pdf]

Supplementary Methods

SIRT1 immunohistochemistry quantification. For renal biopsies, the glomerular areas were delimited on ImageJ software (National Institutes of Health, Bethesda, MD, USA). Next, a color threshold was set: hue value, 0-255; saturation, 0-255; brightness, 10-130. The positive nuclear spots for SIRT1 were counted by analyzing particles. The positive spots/glomerular area ratio was used for statistical analysis.

Claudin-1 immunohistochemistry quantification. For renal biopsies, the glomerular areas were delimited on ImageJ software (National Institutes of Health). Next, a color threshold was set: hue value, 0-255; saturation, 0-255; brightness, 10-140. The positive area values for claudin-1 were used for statistical analysis.

Podocin immunohistochemistry quantification. For renal biopsies, the glomerular areas were delimited on ImageJ software (National Institutes of Health). Next, a color threshold was set: hue value, 0-255; saturation, 0-255; brightness, 10-150. The positive area values for podocin were used for statistical analysis.

SIRT1 immunofluorescence quantification. For mice glomeruli, the glomerular areas were delimited manually on ImageJ software (National Institutes of Health) and cropped. Next, a color threshold was set: hue value, 0-255; saturation, 0-255; brightness, 100-255. The positive area values for SIRT1 were used for statistical analysis.

Claudin-1 immunofluorescence quantification. For mice glomeruli, the glomerular areas were delimited manually on ImageJ software (National Institutes of Health) and cropped. Next, a color threshold was set: hue value, 0-255; saturation, 0-255; brightness, 100-255. The positive area values for claudin-1 were used for statistical analysis.

WT1 immunofluorescence quantification. For mice glomeruli, the positive spots for WT1 and DAPI were counted manually on ImageJ software (National Institutes of Health). The WT1/DAPI ratio was used for statistical analysis.

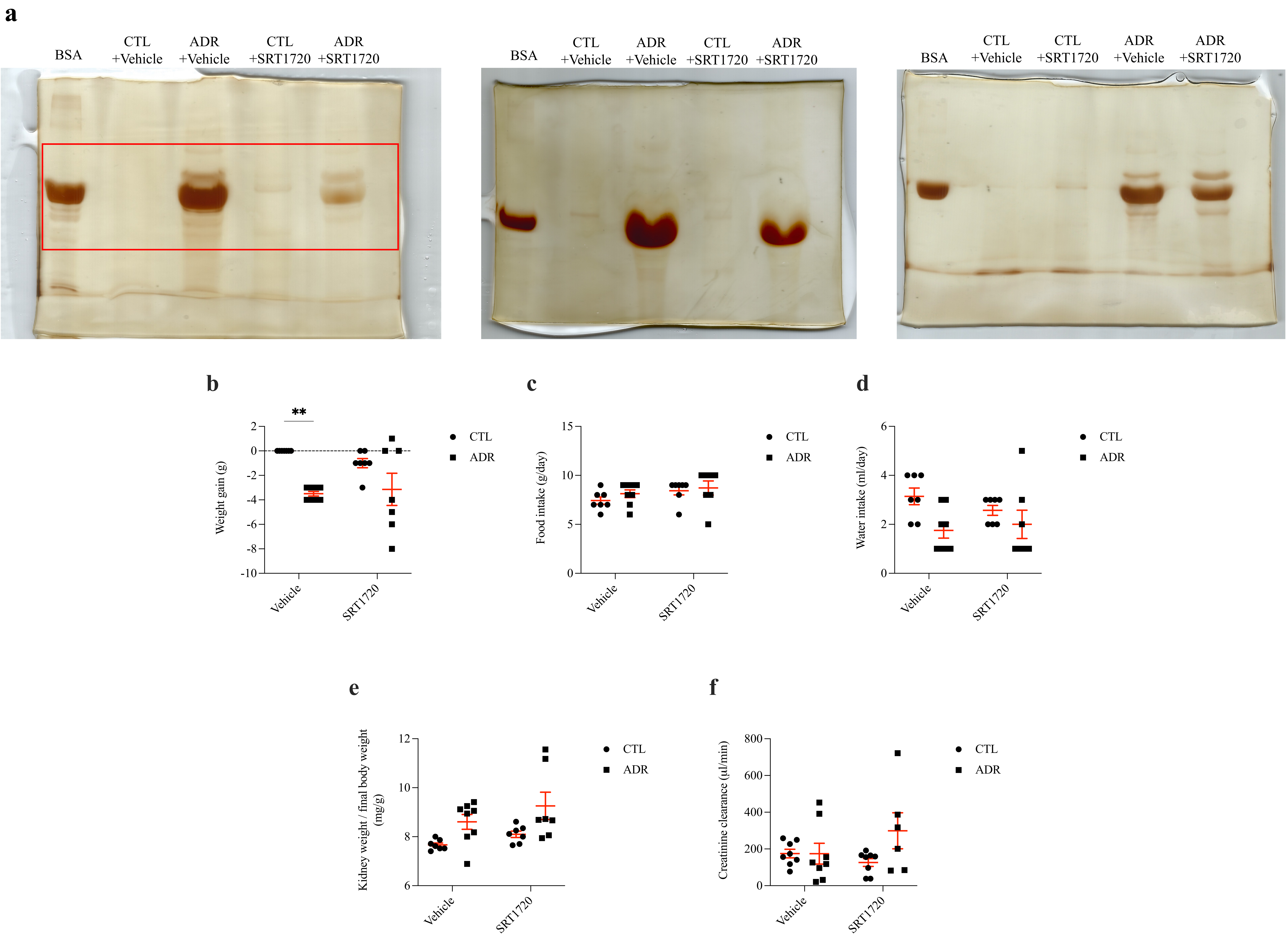

Supplementary Figure S1. Animal experimentation data. (a) 10% SDS-PAGE analysis of 24-hour urine obtained from BALB/c mice. (b) Weight gain. (c) Food intake. (d) Water intake. (e) Kidney weight/final body weight. (f) Creatinine clearance. Statistical analyses were performed by two-way ANOVA. Values were expressed as mean □ SEM. ** $P < 0.01$. CTL, control mice (graph circles); SDS-PAGE, sodium dodecyl sulfate–polyacrylamide gel electrophoresis; ADR, Adriamycin injected mice (graph squares); SRT1720, specific SIRT1 agonist.

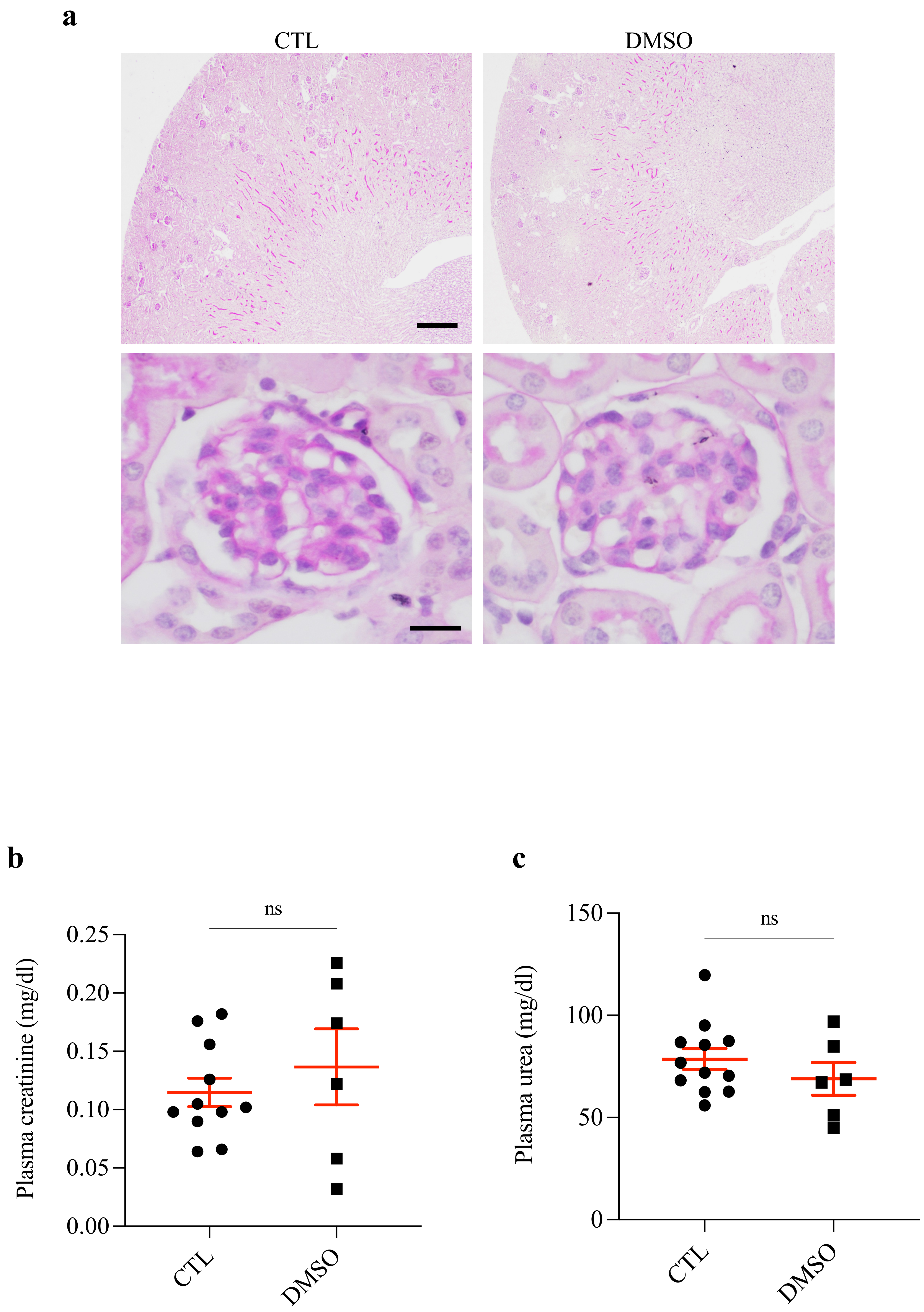

Supplementary Figure S2. Animal experimentation data. (a) Representative PAS micrographs (4 µm-thick) show that DMSO 10% (v/v, vehicle) chronic administration did not affect renal morphology. Scale bar, 100 µm (40x, kidney cortex) and 10 µm (400x, glomerular section). (b) Plasma creatinine analysis. (c) Plasma urea analysis. Statistical analyses were performed by parametric unpaired t-test with Welch's correction. Values were expressed as mean □ SEM. PAS, Periodic acid-Schiff; ns, non-significant.

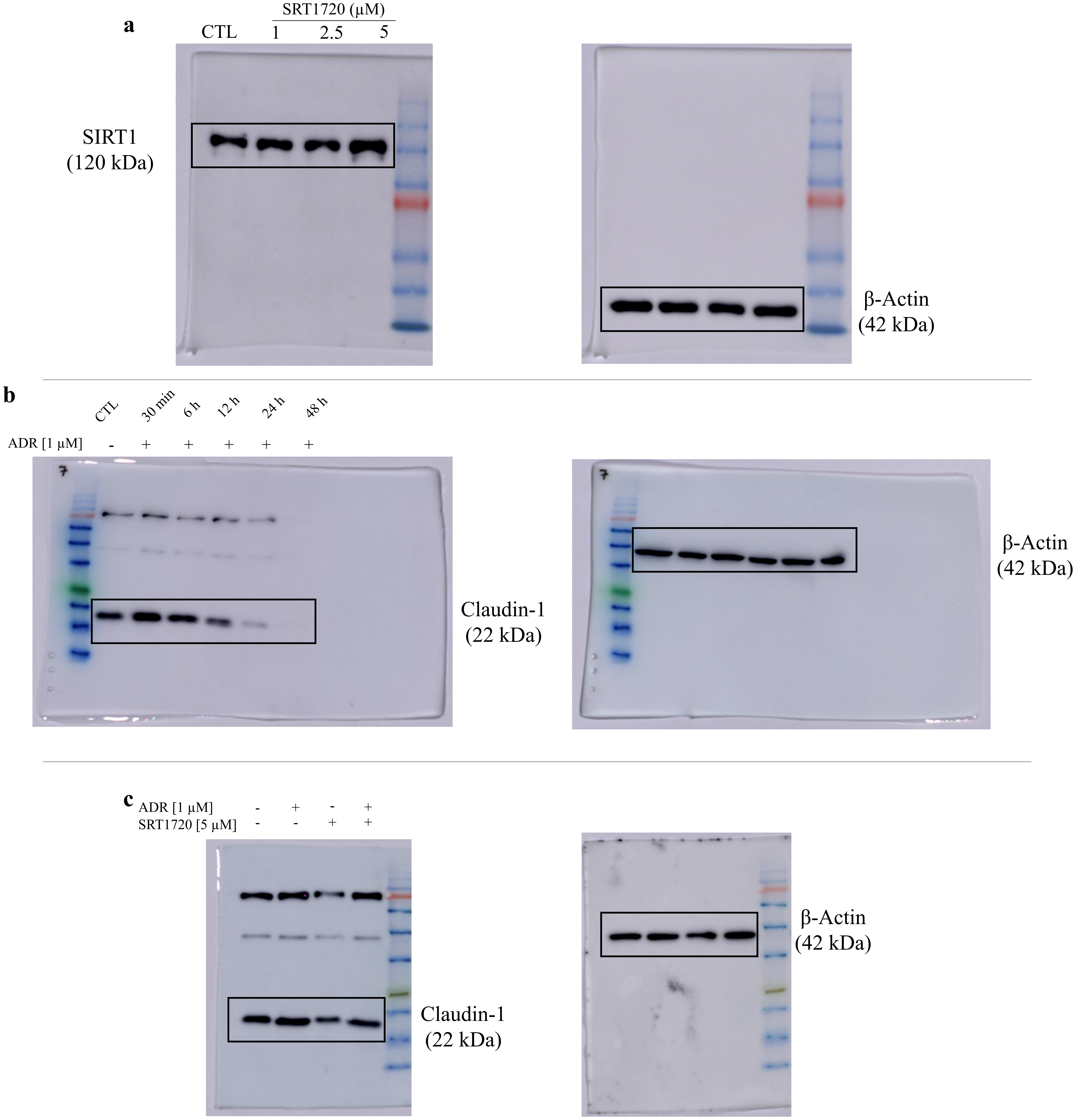

Supplementary Figure S3. Immunoblotting experiments of podocyte cell culture. (a) Full-length blots of SIRT1 after SRT1720 treatment. (b) Full-length blots of claudin-1 in Adriamycin-treated cells. (c) Full-length blots of claudin-1 in SRT1720-pretreated and Adriamycin treated cells.
