# Supplementary material for: Evaluation of glomerular sirtuin-1 and claudin-1 in the pathophysiology of nondiabetic focal segmental glomerulosclerosis
Source: Sci Rep. 2023 Dec 19;13:22685. doi: 10.1038/s41598-023-49861-0 (PMC10730508; doi:10.1038/s41598-023-49861-0)
Supplement: Supplementary file 2 — Supplementary Table S1. [file 41598_2023_49861_MOESM2_ESM.docx]

**Supplementary Table S1*.*** Animal experimentation data.

|  | ***CTL*** | ***10% DMSO*** | ***P value*** |
| --- | --- | --- | --- |
| Plasma urea, mg/dl | 78.6 ± 5.08 | 69.0 ± 8.00 | 0.3358 |
| Plasma creatinine, mg/dl | 0.115 ± 0.012 | 0.137 ± 0.032 | 0.5516 |

Statistical analyses were performed by parametric unpaired t-test with Welch’s correction. Values were expressed as mean ± SEM.
