# Supplementary material for: Evaluation of glomerular sirtuin-1 and claudin-1 in the pathophysiology of nondiabetic focal segmental glomerulosclerosis
Source: Sci Rep. 2023 Dec 19;13:22685. doi: 10.1038/s41598-023-49861-0 (PMC10730508; doi:10.1038/s41598-023-49861-0)
Supplement: Supplementary file 3 — Supplementary Table S2. [file 41598_2023_49861_MOESM3_ESM.docx]

**Supplementary Table S2*.*** Baseline characteristics of patients diagnosed with nondiabetic focal segmental glomerulosclerosis.

| ***Case*** | ***Serum creatinine (mg/dl)*** | ***Proteinuria (mg/dl)*** | ***Hematuria*** |
| --- | --- | --- | --- |
| 1 | NA | NA | NA |
| 2 | 1.8 | 3.98 | Absent |
| 3 | 1.23 | 2.02 | Absent |
| 4 | 0.89 | 1.75 | Absent |
| 5 | 0.85 | NA | NA |
| 6 | 1.04 | NA | NA |
| 7 | 1.33 | 0.31 | Absent |
| 8 | NA | NA | NA |

NA, not available.
